# Successful Treatment of Iron Deficiency Anemia with Ferric Carboxymaltose in an Elderly Patient with Multiple Comorbidities and COVID-19

**DOI:** 10.7759/cureus.16997

**Published:** 2021-08-08

**Authors:** Vincenzo Bassi, Valentina Apuzzi, Francesco Calderaro, Massimo Piroddi

**Affiliations:** 1 U.O.C. di Medicina Generale e Lungodegenza, San Giovanni Bosco Hospital, ASL Napoli 1 Centro, Naples, ITA

**Keywords:** anemia, ferric carboxymaltose, covid-19, hospitalization, elderly

## Abstract

Anemia is frequently associated with older age and comorbidities. Also, anemia is a frequent finding in patients hospitalized for Coronavirus infectious disease 2019 (COVID-19), where it has been associated with poor outcomes. Management of anemia is thus crucial in this setting. We present the case of an elderly woman with chronic iron deficiency anemia and multiple comorbidities, hospitalized for COVID-19, whose iron deficiency was successfully treated with ferric carboxymaltose. Hemoglobin and iron stores were replenished, and transferrin saturation increased to average values. Ferric carboxymaltose was well tolerated, and there were no safety concerns. The patient recovered from COVID-19 was discharged 25 days after admission.

## Introduction

Anemia is associated with older age and several comorbidities such as cardiovascular disease, renal disease, and hypertension. Anemia is also a common finding in patients with Coronavirus infectious disease 2019 (COVID-19), where it has been reported in over 60% of cases and associated with significantly worse outcomes compared to those without anemia [1,2]. Even if IV iron should be used with caution in acute or chronic infection cases, the correct diagnosis and management of iron deficiency anemia are crucial in patients hospitalized with COVID-19 [3].

With the aim of sharing best practices in these fragile patients, we present here the unique case of an elderly woman with chronic iron deficiency anemia and multiple comorbidities, hospitalized for COVID-19, whose iron deficiency was treated with ferric carboxymaltose (Ferinject, Vifor International).

## Case presentation

An 87-year-old woman was transferred to our department (Internal Medicine, San Giovanni Bosco Hospital, Naples) from another hospital (Vecchio Pellegrini Hospital) on Jan 26, 2021, for interstitial pneumonia due to COVID-19. Upon admission, the patient had a computed tomography (CT) score of 3/20 based on Chung and colleagues' criteria [4]. The patient had a significant medical history that included chronic iron deficiency anemia, chronic obstructive pulmonary disease (COPD), hypertension, persistent atrial fibrillation, and chronic cerebral vasculopathy. She was receiving regular therapy with amiodarone, oral ferrous sulfate, ramipril, and aclidinium bromide. Prior to admission, she had received 2 units of packed red blood cells for severe anemia in the emergency department at the other hospital (Hb was 7.2 g/dl).

**Figure 1:**
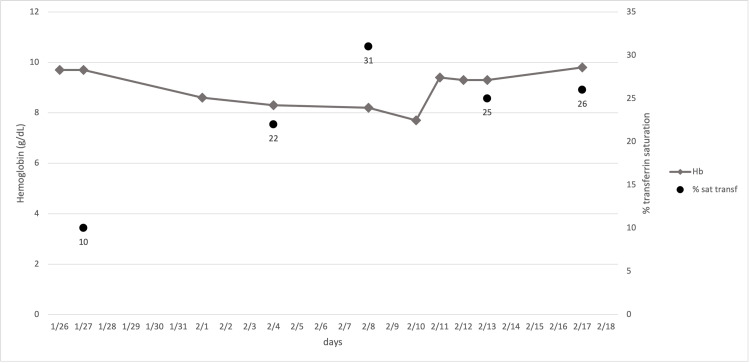
Values of Hb and transferrin saturation during recovery. Administrations of IV Ferric carboxylmaltose were on 1/31 and 02/08.

Upon admission, laboratory studies revealed a hematocrit of 32,7% (38 - 46%), microcytic anemia with hemoglobin 9.7 g/dl (11.7 - 13.8 g/dL), a mean corpuscular volume of 74 fl (80-100fl). Ferritinemia was in the average range of 103 ng/mL (20-120 ng/dL) while transferrin saturation was only 10%. Levels of Hb and transferrin saturation during hospital recovery are shown in Figure 1.

On day 1, inflammatory marker levels of IL-6 (<7) and C-reactive protein (0.5-1 mg/dL) were 20.2 pg/ml and 0.21 mg/dL, respectively. The esophagogastroduodenoscopy (EGDS) was negative, while the colonoscopy showed the presence of hemorrhoids and the search for occult blood was positive. Given the patient’s history of chronic anemia and low transferrin saturation, she was given IV ferric carboxymaltose 1000 mg on day 4. On day 6, her Hb was 8.6 mg/dl, which further decreased to 8.3 mg/dl on day 9, while transferrin saturation improved to 22%. On day 13, transferrin saturation had increased to 31% with a Hb of 8.2 g/dl, and the patient received 500 mg IV ferric carboxymaltose. On day 15, her Hb decreased to 7.7 g/dl, which was followed by a sharp increase to 9.4 g/dl on day 16, reaching 9.8 g/dl on day 22 of admission with a transferrin saturation of 26%. On day 25, given the stable condition of the patient, the negative inflammatory markers (IL-6 and C.reactive protein) and a negative nasopharyngeal swab for SARS-CoV-2, she was discharged from her previous therapeutic regimen with the indication to evaluate anticoagulant therapy with direct-acting oral anticoagulants (DOACs) after appropriate controls of hemoglobin in consideration of a CHADS2-VASc score of 6 and HAS-BLED of 3.

## Discussion

To the best of our knowledge, the present case is the first report of an anemic patient with COVID-19 receiving treatment with ferric carboxymaltose. Irrespective of its etiology, the main objectives for management of iron deficiency anemia include replenishment of iron stores, and normalization of Hb levels and overall iron indices (such as ferritin levels). These adjustments have a positive impact on the patient’s functional capacity and QoL [5,6]. IV iron should be used with caution in acute or chronic infection cases, asthma, eczema, or atopic allergies, and should be discontinued in patients with ongoing bacteremia. However, recent studies have shown no increased risk of infections with IV iron and are in line with our clinical experience [5]. In particular, the PIVOTAL study showed no increased risk of infection with substantial IV iron supplementation in fragile patients under dialysis [7]. In the light of controversial theoretical data about the safety of IV iron in patients with chronic infections, we suggest that a benefit/risk assessment should be performed in order to evaluate each case. In our elderly patient, it is unclear if the worsened anemia was due to increased inflammation. While C-reactive protein and IL-6 were raised, they were not increased to levels associated suggestive of a hyperinflammatory reaction [8]. Moreover, IL-6 levels at discharge were on average indicating a lack of proinflammatory effect of iron. In any case, the patient was deemed a high risk for several factors, which in addition to anemia, included advanced age, hypertension, and COPD [9,10]. Anemia in patients hospitalized for COVID-19 has been associated with mortality as well as the need for invasive ventilation and ICU admission [2]. Adequate management of anemia in patients hospitalized for COVID-19 in this group of patients is thus warranted. Our patient had previously received an emergency transfusion prior to admission, which improved Hb levels. Blood supplies have been severely interrupted during the COVID-19 pandemic [3,11]. Even if serum ferritin levels were considered in the average range in our patient, ongoing therapy for iron was deemed necessary as transferrin saturation was < 20%, which is a recently proposed threshold used to diagnose iron-deficiency anemia during inflammation [12]. Several data have highlighted that during inflammation, the sole interpretation of serum ferritin levels could be not enough as hepcidin and apoferritin levels tend to be higher, the first inducing iron sequestration in macrophages [12].

Ferric carboxymaltose is a colloidal solution of nanoparticles consisting of a polynuclear iron (III)-(oxyhydr)oxide core that is stabilized by carboxymaltose, which may be given as a single high-dose, 15-min infusion [6]. Intravenous administration has been shown to provide a high iron bioavailability, and it is well tolerated in patients with a wide range of comorbidities [6]. Ferric carboxymaltose thus seemed a suitable choice to correct iron depletion in our patient and allowed us to infuse 1000 mg of iron in a short time frame. It is also better tolerated than oral ferrous sulfate, which is associated with adverse gastrointestinal events and bowel microbiome changes [13]. We observed no adverse effects of ferric carboxymaltose in our patient, which corrected the anemia and increased transferrin saturation to average values. However, it is important to underline that clinical data on different IV iron products should not be assumed to be equivalent, unless compared in head-to-head studies, due to their different physiochemical properties and pharmacokinetic/pharmacodynamic profiles [14]. In particular, ferric carboxymaltose is characterized by a low labile iron content, which seems to stimulate the growth of microorganisms [5,14]. Use of ferric carboxymaltose for iron deficiency after discharge for heart failure has recently been reported in a large trial involving 1132 patients, showing a significant benefit of ferric carboxymaltose on a combined endpoint of heart failure hospitalizations and cardiovascular death [15]. The trial is of interest as it is one of the first randomized clinical trials reporting results in which outcomes were potentially affected by the COVID-19 pandemic. However, the authors could not predict what influence COVID-19 might have had on treatment, even if benefits were still seen in a pre-COVID-19 analysis [15].

## Conclusions

Ferric carboxymaltose (Ferinjectâ, Vifor International) was used to treat iron deficiency anemia in an elderly patient with multiple comorbidities and hospitalized for COVID-19. The treatment led to rapid replenishment of iron stores, normalization of Hb, and concomitant improvement in transferrin saturation. Ferric carboxymaltose was well tolerated, and there were no safety issues. Thus, in our patient, the treatment exhibited an excellent risk/benefit profile while most importantly, made it possible to avoid other transfusions and did not influence the course of the infection. This highlights that ferric carboxymaltose can be considered as a valid option to correct anemia in the setting of hospitalization for COVID-19 and avoid transfusion regimens, as in severe patients with diffuse coagulopathy such as in the present case.

## References

[1] Bergamaschi G, Borrelli de Andreis F, Aronico N (2021). Anemia in patients with Covid-19: pathogenesis and clinical significance. Clin Exp Med.

[2] Dinevari MF, Somi MH, Majd ES, Farhangi MA, Nikniaz Z (2021). Anemia predicts poor outcomes of COVID-19 in hospitalized patients: a prospective study in Iran. BMC Infect Dis.

[3] Stanworth SJ, New HV, Apelseth TO (2020). Effects of the COVID-19 pandemic on supply and use of blood for transfusion. Lancet Haematol.

[4] Chung M, Bernheim A, Mei X (2020). CT imaging features of 2019 novel Coronavirus (2019-nCoV). Radiology.

[5] DeLoughery TG (2019). Safety of oral and intravenous iron. Acta Haematol.

[6] Scott LJ (2018). Ferric carboxymaltose: a review in iron deficiency. Drugs.

[7] Macdougall IC, White C, Anker SD (2019). Intravenous iron in patients undergoing maintenance hemodialysis. N Engl J Med.

[8] Lavillegrand JR, Garnier M, Spaeth A (2021). Elevated plasma IL-6 and CRP levels are associated with adverse clinical outcomes and death in critically ill SARS-CoV-2 patients: inflammatory response of SARS-CoV-2 patients. Ann Intensive Care.

[9] Kang SJ, Jung SI (2020). Age-related morbidity and mortality among patients with COVID-19. Infect Chemother.

[10] Lee SC, Son KJ, Han CH, Park SC, Jung JY (2021). Impact of COPD on COVID-19 prognosis: a nationwide population-based study in South Korea. Sci Rep.

[11] Okocha O, Luan Erfe BM, Peace JM, Sweitzer J (2020). Intravenous iron to treat anemia becomes an essential service to conserve blood during the COVID-19 pandemic. J Clin Anesthesia Pain Management.

[12] Cappellini MD, Musallam KM, Taher AT (2020). Iron deficiency anaemia revisited. J Intern Med.

[13] Keating GM (2015). Ferric carboxymaltose: a review of its use in iron deficiency. Drugs.

[14] Martin-Malo A, Borchard G, Flühmann B, Mori C, Silverberg D, Jankowska EA (2019). Differences between intravenous iron products: focus on treatment of iron deficiency in chronic heart failure patients. ESC Heart Fail.

[15] Ponikowski P, Kirwan BA, Anker SD (2020). Ferric carboxymaltose for iron deficiency at discharge after acute heart failure: a multicentre, double-blind, randomised, controlled trial. Lancet.

